# Study of Impact Damage in PVA-ECC Beam under Low-Velocity Impact Loading Using Piezoceramic Transducers and PVDF Thin-Film Transducers

**DOI:** 10.3390/s18020671

**Published:** 2018-02-24

**Authors:** Baoxin Qi, Qingzhao Kong, Hui Qian, Devendra Patil, Ing Lim, Mo Li, Dong Liu, Gangbing Song

**Affiliations:** 1 College of Civil Engineering, Shenyang Jianzhu University, Shenyang 110168, China; qibaoxin2005@163.com; 2 Department of Mechanical Engineering, University of Houston, Houston, TX 77204, USA; deven1p@gmail.com (D.P.); dongliu@uh.edu (D.L.); gsong@central.uh.edu (G.S.); 3 College of Civil Engineering, Zhengzhou University, Zhengzhou 450001, China; chansteed@126.com; 4 Department of Civil and Environmental Engineering, University of Houston, Houston, TX 77204, USA; ilim@uh.edu; 5 Department of Civil and Environmental Engineering, University of California, E4145 Engineering Gateway, Irvine, CA 92617, USA; mo.li@uci.edu

**Keywords:** impact damage detection, PVA-ECC beam, piezoceramic transducers, PVDF transducers, active sensing approach, short-time Fourier transform, smart aggregate

## Abstract

Compared to conventional concrete, polyvinyl alcohol fiber reinforced engineering cementitious composite (PVA-ECC) offers high-strength, ductility, formability, and excellent fatigue resistance. However, impact-induced structural damage is a major concern and has not been previously characterized in PVA-ECC structures. We investigate the damage of PVA-ECC beams under low-velocity impact loading. A series of ball-drop impact tests were performed at different drop weights and heights to simulate various impact energies. The impact results of PVA-ECC beams were compared with mortar beams. A combination of polyvinylidene fluoride (PVDF) thin-film sensors and piezoceramic-based smart aggregate were used for impact monitoring, which included impact initiation and crack evolution. Short-time Fourier transform (STFT) of the signal received by PVDF thin-film sensors was performed to identify impact events, while active-sensing approach was utilized to detect impact-induced crack evolution by the attenuation of a propagated guided wave. Wavelet packet-based energy analysis was performed to quantify failure development under repeated impact tests.

## 1. Introduction

Accelerated economic development across the world has increased the need and number of large-scale bay bridges and cross-sea bridges, as well as the ships and shipping routes that travel across these bridges. This has resulted in an increased number of impact collision accidents between ships and bridges which is a great source of economic loss. In structural terms, such impact accidents may cause uncoordinated deformation and fractures of expansion joints in bridges [1,2], and may even result in bridge collapse [3,4].

Research on structural impact detection has been ongoing for many years [5,6,7,8]: Kim et al. [9,10] developed an iterative regularization method to reconstruct the impact force on a graphite/epoxy platform using polyvinylidene fluoride (PVDF) sensors. Wu et al. [11] subsequently presented an optimized technique which identified the impact force and estimated the impact location for isotropic plates by using strain gauge signals, and Shih et al. [12] studied the impact process based on the spectral analysis of acoustic emission signals. Likewise, research on impact-induced damage have also been intensely studied in recent years [13,14,15]: Santosa et al. [16] detected the impact damage on fiberglass composite plates based on surface lava wave propagation, and Kim et al. [17] utilized wavelet-based approach and short-time Fourier transform (STFT) to detect impact damage in composite laminates. Lastly, impact damage detection in concrete structures has become a focus in recent research: Song et al. [18] monitored and evaluated impact damage on bridges due to high-speed vehicle collision using embedded piezoceramic transducers. Demi et al. [19] also embedded piezoceramic transducers in concrete structures, and devised a structural damage indication and quantification method based on root-mean-square deviation method. Jeon et al. [20] investigated the low velocity impact and delamination buckling behavior of composite laminates with embedded optical fibers. Minakuchi et al. [21] developed a fiber-optic-based sensing system to monitor the impact damage of a large-scale Carbon Fiber Reinforced Polymer (CFRP) structure. Based on comparative vacuum monitoring (CVM), and Brillouin-based distributed strain measurement, damage areas can be effectively identified. 

Engineering cementitious composites (ECC) has gained popularity in infrastructure construction due to its unique cracking behavior [22], high toughness and energy absorption ability [23], which is approximately 500 times larger than conventional concrete or fiber-reinforced concrete [24], and better shearing resistance [25]. In 1998, Li et al. [26] developed polyvinyl alcohol fiber reinforced engineering cementitious composite (PVA-ECC) with a high tensile strain capacity [27], high compressive strength at early stage [28], and a strong ability to absorb energy [29,30,31]. The high tensile performance of PVA-ECC enables the material to be used as a cast-in-place bridge panel [32], and the high energy absorption characteristics makes PVA-ECC desirable to counter seismic effects on high-rise buildings [33,34]. Furthermore, PVA-ECC can also be used to repair the surface of retaining walls, channels, and viaducts [35]. Thus, PVA-ECC materials have become widespread in bridges and large infrastructure developments. However, the study of impact-induced damage on PVA-ECC structures has not garnered widespread study; specifically, the monitoring of crack formation and development of PVA-ECC beams and columns under impact loads lack detailed studies.

In this paper, we investigate the impact damage of PVA-ECC beams under low-velocity impact loading. Two types of beam specimens—PVA-ECC and mortar beams—were fabricated and compared in the laboratory. For each beam, one PVDF thin-film sensor and a pair of smart aggregate were preinstalled at predetermined locations within the beam before casting. A series of impact tests at various impact energies were conducted, impact energies were varied by changing the drop weight and height. The impact events were recorded by using short-time Fourier transform (STFT) of the signal received by the PVDF thin-film sensors. Guided transmission waves were propagated between the smart aggregate pair, and the attenuation of the propagated signal was used to monitor the evolution of the impact-induced crack. Furthermore, the signal received by the smart aggregate was computed using wavelet packet-based energy approach to offer a quantitative indicator to the crack evolution under repeated impact tests.

## 2. Embedded PVDF Thin-Film Sensor and Smart Aggregate

### 2.1. Constitutive Equations of Piezoelectric Material

Piezoelectric materials generate strain or stress when an electrical field is applied, and vice versa. Lead Zirconate Titanate (PZT) and PVDF are two of the commonly used piezoelectric materials. Due to the improved strain flexibility, impact resistance, and lower permittivity coefficient of PVDF over PZT, the PVDF sensor is more suitable to be used as impact sensors to measure the structural impact events. On the other hand, PZT generates relatively larger strain or stress than PVDF when the electrical field is applied, so that PZT materials are more suitable to be utilized as actuators in the active sensing approach. In this research, embedded PVDF thin-film sensors are designed for monitoring the impact events and PZT-based smart aggregate (SA) pair was deployed to the specimen to perform active sensing. The constitutive relations for piezoelectric materials such as PZT and PVDF are given as,

(1) $$\left[\begin{array}{l} D\\ S \end{array}\right]=\left[\begin{array}{cc} \bar{\varepsilon^{T}} & d\\ d^{T} & \bar{s^{E}} \end{array}\right]\left[\begin{array}{c} E\\ T \end{array}\right]$$

where *D*(3 × 1) is electric displacement vector; *S*(6 × 1) is strain vector; *E*(3 × 1) is the applied external electric field vector; *T*(6 × 1) is the stress vector; $\bar{\varepsilon_{ij}^{T}}=\varepsilon_{ij}^{T}(1-\delta j)(3\times 3)$ is the matrix of the complex dielectric permittivity at constant stress; $\bar{s_{km}^{E}}=s_{km}^{E}(1-\eta j)(6\times 6)$ is the matrix of the complex elastic compliance at constant electric field; $d_{im}(3\times 6),d_{jk}^{T}(6\times 3)$ are the matrices of the piezoelectric strain coefficients; δ is the dielectric loss factor; and η is the mechanical loss factor.

Considering the PVDF sensor and PZT-based SA sensor are embedded in the specimen, they can be simplified as a beam subjected to bending with regarding its size. Meanwhile, for bending elements, the piezoelectric material is poled along the 3 axis, as shown in Figure. Given these assumptions, only a one-dimensional stress will be generated along the 3 axis in PVDF-based sensor and PZT-based SA sensor. Then, the constitutive relations can be simplified as,

(2) $$\left\{ \begin{array}{l} D_{3}=d_{33}T_{3}+\varepsilon_{3}^{T}E_{3}\\ S_{1}=s_{33}^{E}T_{3}+d_{33}E_{3} \end{array}\right.$$

Furthermore, because PVDF material is used as sensor in this paper, the applied external electric field, viz., *E*_3_ is zero. Therefore, the generated electrical charge of PVDF thin-film sensor can be expressed as,

(3) $$q=\int E_{p}d_{33}S_{3}dA$$

where $E_{p}=1/S_{11}^{E}$ is the elasticity modulus of PVDF; *A* is the electrode area in the 1–2 plane.

**Figure 1:**
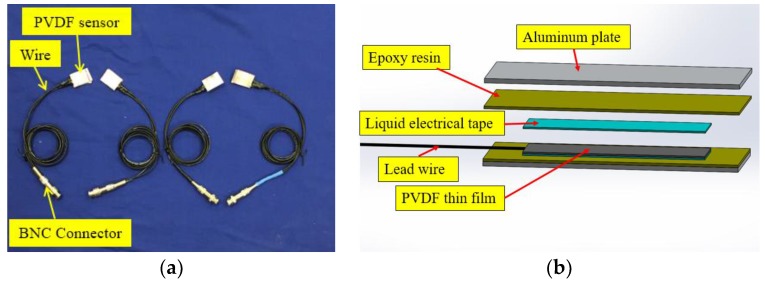
Embedded polyvinylidene fluoride (PVDF) thin-film sensor: (**a**) photo and (**b**) internal structure.

In this research, embeddable PVDF thin-film sensors were designed to detect the impact events, as shown in Figure 1. The PVDF thin-film sensors used in this experiment consists of two thickness of 2-mm aluminum plate and the middle PVDF film thickness is 0.05 mm. The PVDF thin-film sensor was connected by lead wires and isolated by liquid electrical tape. Subsequently, two aluminum plates house the sensor, and the entire module is stabilized with epoxy resin. The aluminum housing protects the PVDF thin-film from physical and chemical damage, as well as load imbalance when embedded within concrete structure. The detailed mechanical and material properties of PVDF and PZT transducers used in this research is given in Table 1.

### 2.2. Short-Time Fourier Transform

The short-time Fourier transform (STFT), is a Fourier-related transform which can be used in time-frequency analysis. Based on the window function, the signal can be divided into small windows. Therefore, the local spectral density of the signal in each window can be calculated. The STFT can be defined as

$$\mathrm{STFT}\;\left(\tau ,\omega \right)=\int_{-\infty }^{+\infty }x\left(t\right)g\left(t-\tau \right)e^{-i\omega t}\mathrm{dt}$$

where g(t) represents the window function, and x(t) is the original signal to be transformed. τ is the time index that can adjust the resolution of the time axis. In this research, STFT was used to analyze the received signal of the PVDF sensor due to the impact energy. Based on the STFT technique, the time information, frequency information, as well as the impact energy information can be obtained. 

### 2.3. Smart Aggregate-Based Active Sensing Approach

**Figure 2:**
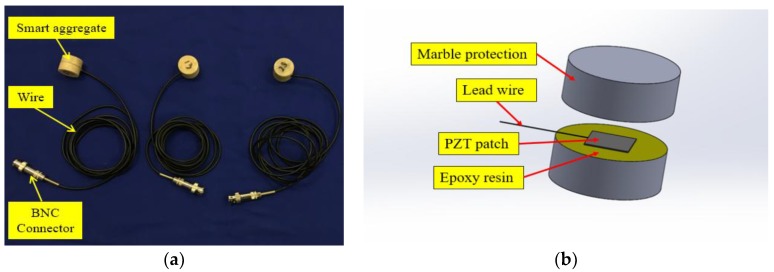
Smart aggregate (**a**) photo and (**b**) internal structure.

To evaluate the severity of damage of a concrete beam under discrete repeated impacts, the smart aggregate-based active sensing approach was employed in this research. Smart aggregate (SA) was designed by sandwiching a waterproofed PZT patch with two marble pieces, as shown in Figure 2. The Piezoelectric SA in this experiment is composed of two pieces of granite with 12 mm thickness, and a PZT sheet with a thickness of 0.3 mm in the middle of the smart aggregate. The cylindrical shaped smart aggregate has a radius of 25 mm and a height of 25 mm. In the active sensing approach, one SA was used as an actuator to generate guided waves, while the other SA was used as a sensor to detect the propagated wave response. When impact-induced cracks develop in concrete, the cracks function as stress relief which attenuates the wave propagation energy. The attenuation increases with the degree of failure, therefore the energy received by the SA sensor is an effective indicator to determine the degree of impact-induced cracks in concrete beams. 

### 2.4. Wavelet Packet-Based Signal Energy Analysis

Wavelet packet analysis is widely used in signal processing, image processing, quantum mechanics, and theoretical physics. Applications of wavelet packet-based damage characterization in structural health monitoring have been investigated in recent years. In this research, the total energy of signal was computed by wavelet packet tools. Based on the wavelet decomposition, the energy of each wavelet packet *Ej* (*j* represents the decomposed wavelet packets) can be computed. Therefore, the total energy of the signal can be computed by the energy summation of all the wavelet packets. The total energy of the signal can be expressed as:

(4) $$E=\sum_{l=1}^{l=j}E_{l}$$

By the wavelet packet-based approach, the total energy of the received signal was obtained directly. In our impact tests, the energies of the SA sensor signal of each impact event were computed, and the severity of impact-induced cracks were analyzed based on the computed energy.

## 3. Experimental Setup

### 3.1. Concrete Beam Specimen Fabrication

**Figure 3:**
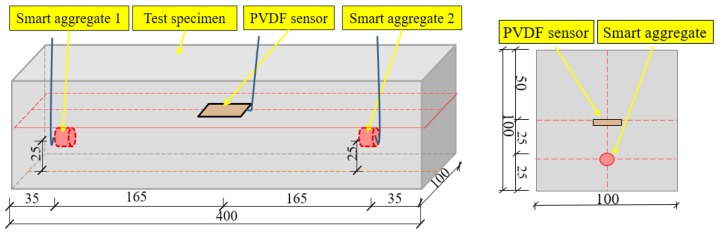
Detailed sensor location (unit: mm).

**Figure 4:**
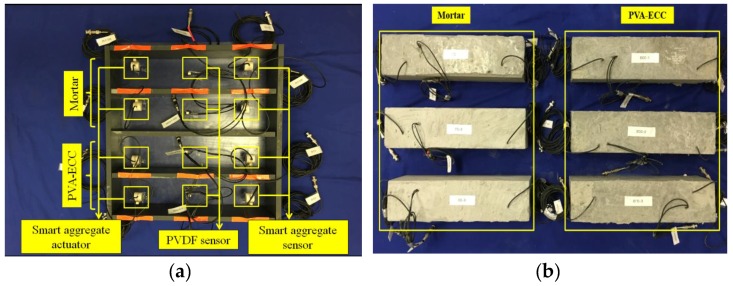
Specimen preparation. (**a**) molds with pre-installed PVDF sensors and smart aggregates (**b**) casted PVA-ECC and mortar beams.

To investigate the impact loading behavior of PVA-ECC and mortar beams, a total of three PVA-ECC and three mortar beams were fabricated in the laboratory. The beams measured 400 × 100 × 100 mm, and the compositions of the beams are shown in Table 2. The detailed properties of PVA fibers are shown in Table 3. Within each beam, one PVDF thin-film sensor and two smart aggregates were positioned at predetermined locations with wooden sticks within the beam molds before casting, as shown in Figure 3 and Figure 4. A PDVF thin-film sensor was used for impact monitoring, and the PVDF film sensor was embedded in the location that is 1/2 thickness away from the surface of the beam to prevent the impact damage to the PVDF thin-film sensor. The crack of PVA-ECC beams is produced in the region of the maximum bending deformation under low-velocity impact loadings, and this region is near the center of the beam. To detect the crack of the PVA-ECC beams, a pair of SAs was embedded in the locations on both sides of the beams, so that to ensure the impact induced cracks occur between the SA pair. To minimize the effect of the sensor wires on the mechanical properties of the specimens, all the wires were placed in straight and come out from the top surface of the specimens. A mechanical vibrator was used during casting for concrete compaction, and the beams were subsequently cured for twenty-eight days.

### 3.2. Low-Velocity Impact Test Setup

**Figure 5:**
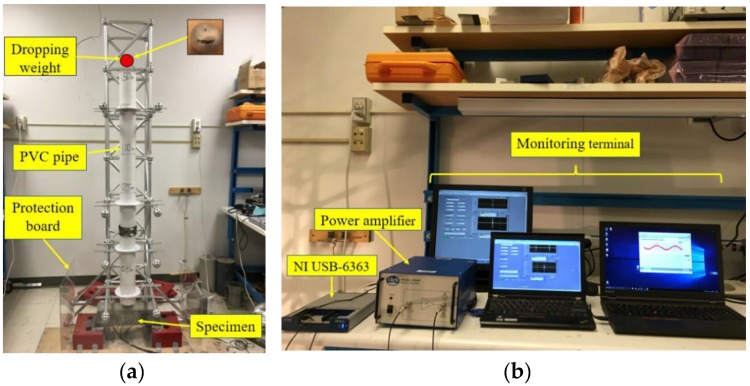
Low-velocity impact test setup (**a**) test setup; (**b**) data acquisition system.

The low-velocity impact test setup is shown in Figure 5. An aluminum frame was used to position PVC pipes which guided a dropping weight to impact on a desired location of the beam. The inner diameter of the pipe measured 10 cm, and three drop heights were used (0.5, 1, and 1.5 m). Two lead ball drop weights (2.724 kg and 8.172 kg) were used. The position of the impact was at the center of the beam. The test beam was fixed onto four L-shaped fixtures which prevented horizontal movement of the beam. The data acquisition system included an NI-6363 data acquisition board, a power amplified, and corresponding monitoring terminals. During each impact test, the signal received by the PVDF sensor was first recorded, then, active sensing with the two smart aggregates was performed. The original propagated signal was a swept sine wave, with parameters provided in Table 4. The signal was amplified 50× by a power amplifier and propagated by the SA actuator. The data acquisition system subsequently recorded the propagated signal response from the SA sensor. The sampling rates of the data acquisition system for the PVDF sensor and SA are 1 kHz and 2 MHz, respectively. A high-resolution camera was used to image crack evolution after each impact test.

## 4. Experimental Procedures

In this test, the free falling impact test was used; the initial velocity is zero. Along with the height increases, the impact velocity of the drop ball is increased. Comparative study on the PVA-ECC beam and Mortar beam under low-velocity impact loading, the influence of drop height and drop weight were investigated. For PVA-ECC beam impact tests, two drop weights and three drop heights were investigated. For PVA-ECC-1, a drop weight of 2.724 kg and drop height of 1.5 m was used. For PVA-ECC-2, the drop weight remained the same while the drop height was decreased to 1 m. For PVA-ECC-3, the drop weight increased to 8.172 kg and the drop height back to 1.5 m. For mortar beam impact tests, only the drop height was varied from 0.5 to 1.5 m. For each beam, impact tests were repeated until complete failure was observed. Due to the lower impact resistance of mortar beam specimens, the drop height for Mortar-3 was reduced to 0.5m. The details of the impact tests are given in Table 5. 

## 5. Results and Discussion

### 5.1. Impact Detection

The impact tests were repeated until each beam completely failed due to impact-induced crack. The total number of impact events for each beam is listed in Table 6. 

#### 5.1.1. PVA-ECC Beams

**Figure 6:**
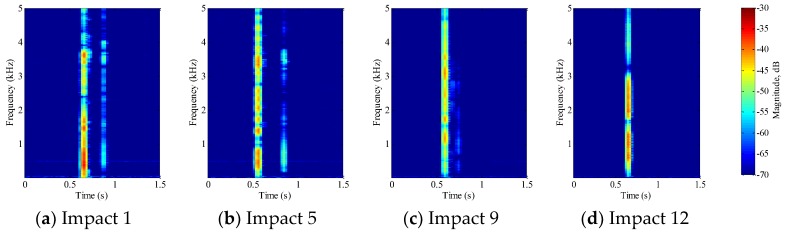
Short-time Fourier transform (STFT) of PVDF sensor signal for PVA-ECC 1.

**Figure 7:**
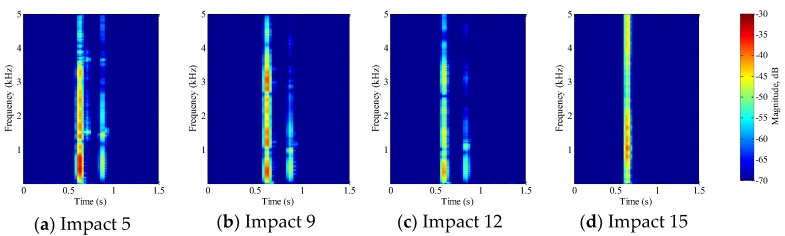
STFT of PVDF sensor signal for PVA-ECC 2.

**Figure 8:**
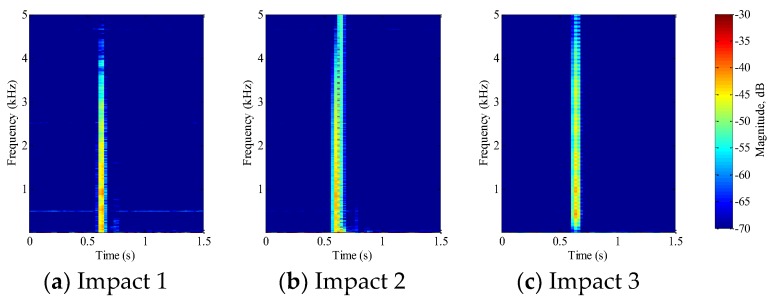
STFT of PVDF sensor signal for PVA-ECC 3.

The impact events detected by the embedded PVDF thin-film sensor in the three PVA-ECC beams are shown in Figure 6, Figure 7 and Figure 8 respectively. The figures represent different stages in the deterioration of the impact-induced crack, and rightmost figures represents the complete failure of the specimen. Complete failure in this research means that the specimen suffers a fracture under the low-velocity impact loading. Within each figure, the impact time and the magnitude versus frequency of impact energy can be obtained by STFT. In addition, smaller impact events were seen immediately following the main impact events in PVA-ECC-1 and 2, which indicated that PVA-ECC specimens exhibited a bouncing effect. The bouncing effect causes free-falling lead balls to bounce up from the initial impact and cause a second smaller impact. However, the bouncing effect weakened as the impact-induced crack increased. As the PVA-ECC beam approached its ultimate bearing capacity, the bouncing effect ceased, as shown in Figure 6d and Figure 7d. This observation implied that a failed PVA-ECC beam structure loses its bouncing effect. The frequency axis shows that the impact energy contributed to a range of impact frequencies. However, the magnitude versus frequency in these Figures does not present a clear trend, which is further elaborated in Section 5.3. For PVA-ECC-3, the drop weight and height were both increased and the beam failed after three impacts, therefore the bouncing effect was not observed in PVA-ECC-3.

#### 5.1.2. Mortar Beams

**Figure 9:**
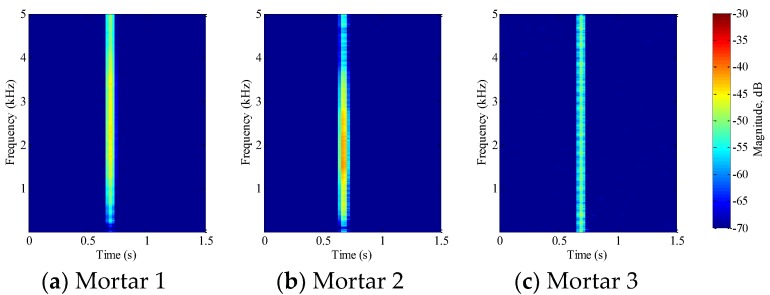
STFT of PVDF sensor signal for PVA-ECC 3.

For mortar beams, each beam completely failed after the first impact test. Figure 9 shows the STFT of the impact signal for each beam. When compared to PVA-ECC beams, mortar beams are prone to fail at the impact loading. With the same drop weight and height, PVA-ECC-1 survived until the 12th impact loading, while the mortar beams failed upon the first impact loading. Even with a reduced drop height of 0.5 m, the mortar beam was still incapable of bearing its integrity under this impact energy.

### 5.2. Crack Detection

#### 5.2.1. PVA-ECC Beams

**Figure 10:**
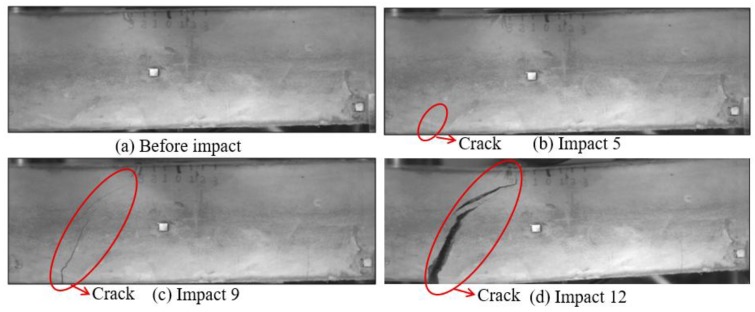
Results of crack detection in PVA-ECC-1 with high-speed video.

**Figure 11:**
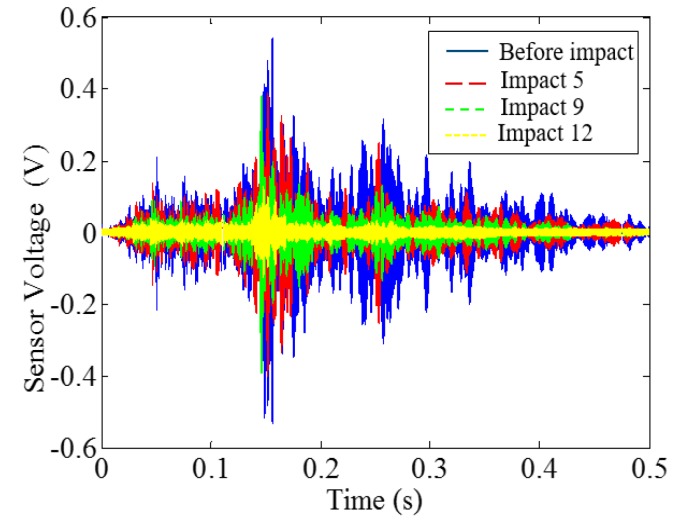
Time-domain signal received by smart aggregate (SA) sensor in PVA-ECC 1.

**Figure 12:**
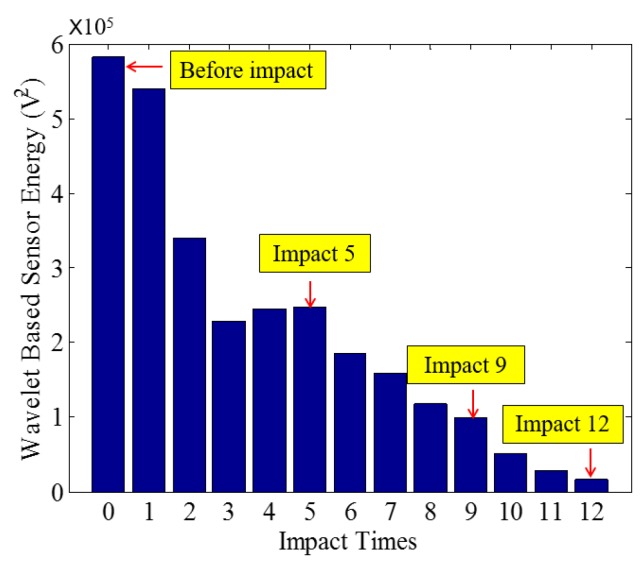
Wavelet packet-based energy plots of the received signal in PVA-ECC 1.

**Figure 13:**
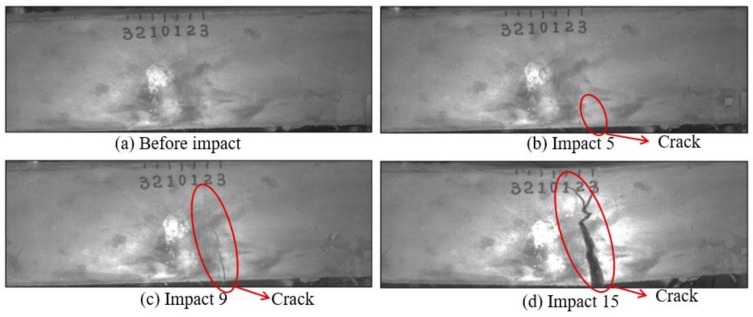
Results of crack detection in PVA-ECC-2 with high-speed video.

**Figure 14:**
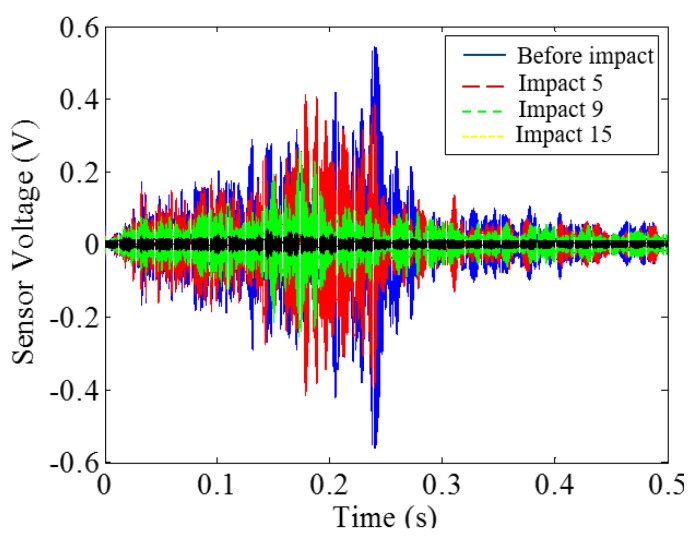
Time-domain signal received by SA sensor in PVA-ECC-2.

**Figure 15:**
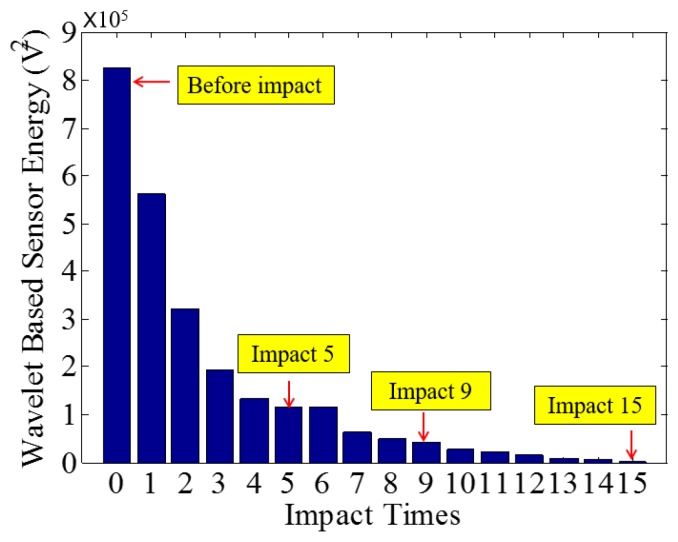
Wavelet packet-based energy plots of the received signal in PVA-ECC-2.

**Figure 16:**
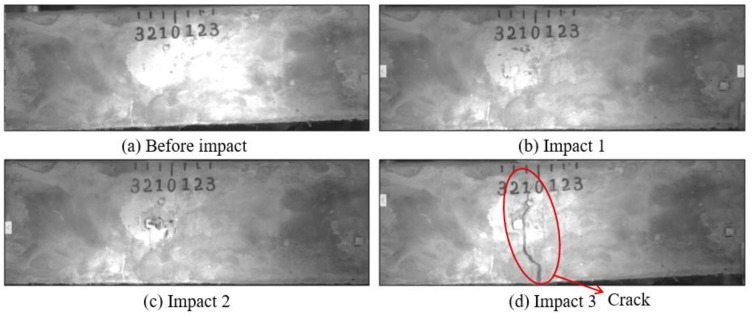
Results of crack detection in PVA-ECC-3 with high-speed video.

**Figure 17:**
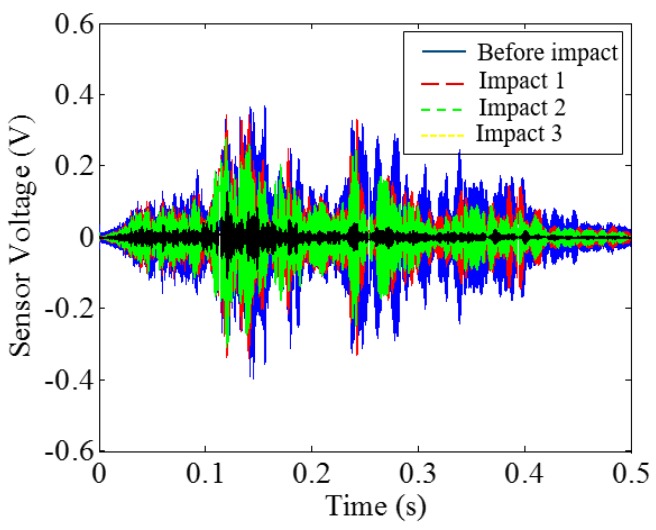
Time-domain signal received by SA sensor in PVA-ECC-3.

**Figure 18:**
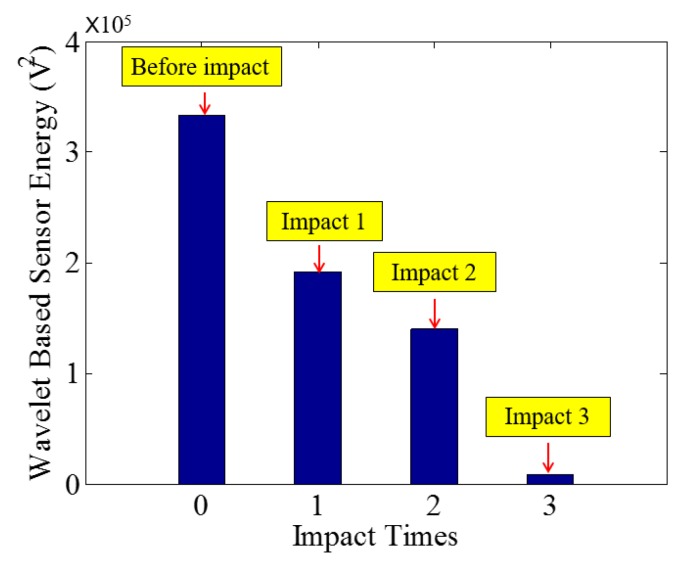
Wavelet packet-based energy plots of the received signal in PVA-ECC-3.

Low-velocity impact tests were repeated for each beam until the beam completely failed. For PVA-ECC-1 to 3, the impact counts were 12, 15, and 3, respectively. Figure 10, Figure 11 and Figure 12 show the PVA-ECC-1 beam after impact tests, the time-domain signal received by the SA sensors, and the signal energy based on wavelet packet-based analysis. Due to space limitation, photos and time-domain signals are shown before impact, after impact 5, after impact 9, and after the last impact for each beam. Evolution of cracks for each beam can be observed. Figure 13, Figure 14, Figure 15, Figure 16, Figure 17 and Figure 18 show the same measurements for PVA-ECC-2 and 3.

Between PVA-ECC-1 and 2, the drop height decreased from 1.5 to 1 m, which resulted in the impact test count increasing from 12 to 15. Since the crack acts as a stress relief, the propagated wave energy decreased which resulted in a decreased signal amplitude as received by the SA sensor. In both their time-domain signal response shown in Figure 6 and Figure 7, it can be seen that the general amplitudes of the received signal decreased as the impact tests continued. In addition, wavelet packet-based signal energy plots provided a quantitative view to present the loss of the received signal energy. When the beam completely failed, the energy of the received signal approached 0 since the stress wave cannot propagate through a major crack. Compared with PVA-ECC-1, the drop weight of PVA-ECC-3 increased from 2.724 kg to 8.172 kg, which resulted in the impact test count decreasing from 12 to 3.

#### 5.2.2. Mortar Beams

**Figure 19:**
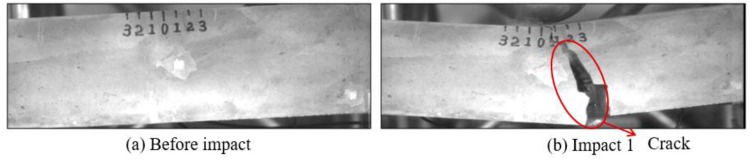
Results of crack detection of Mortar-1 with high-speed video.

**Figure 20:**
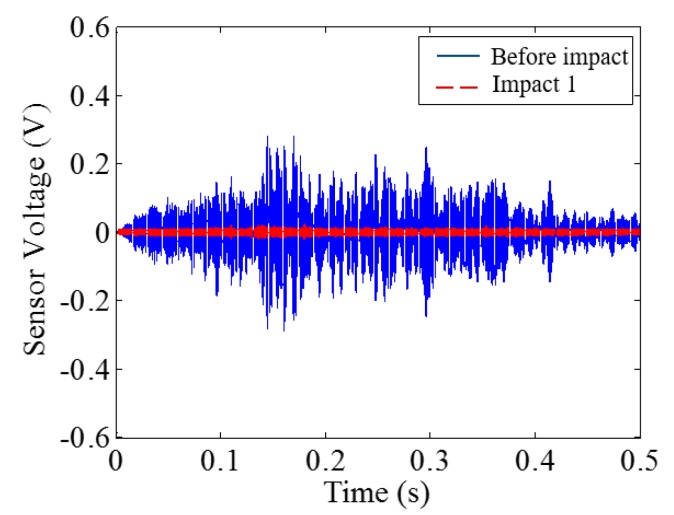
Time-domain signal received by SA sensor in Mortar-1.

**Figure 21:**
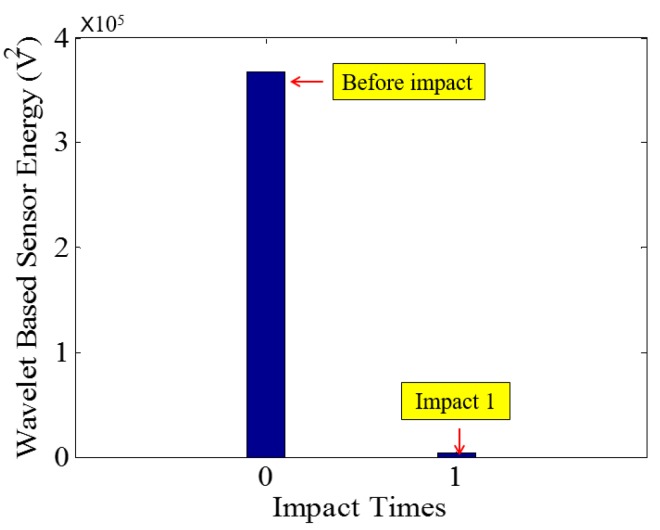
Wavelet packet-based energy plots of the received signal in Mortar-1.

**Figure 22:**
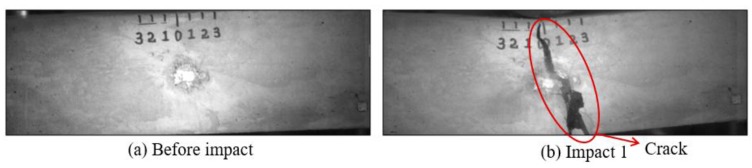
Results of crack detection of Mortar-2 with high-speed video.

**Figure 23:**
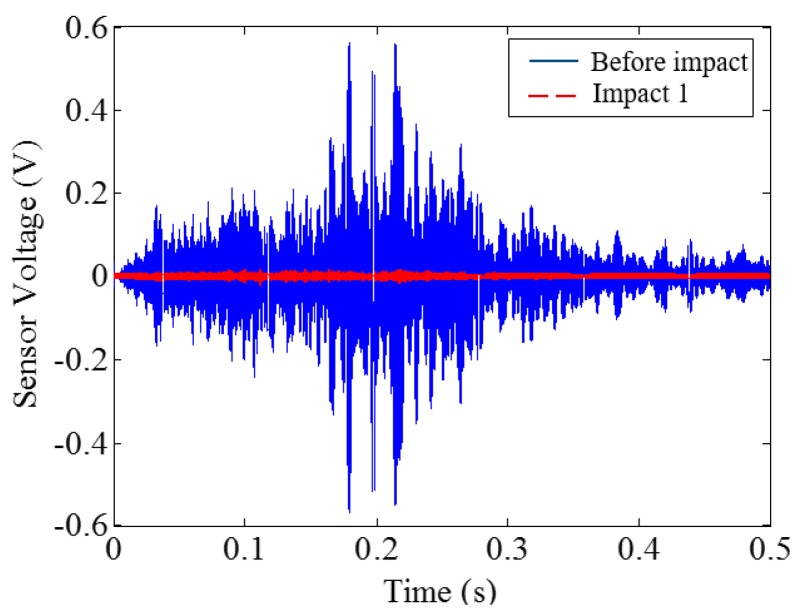
Time-domain signal received by SA sensor in Mortar-2.

**Figure 24:**
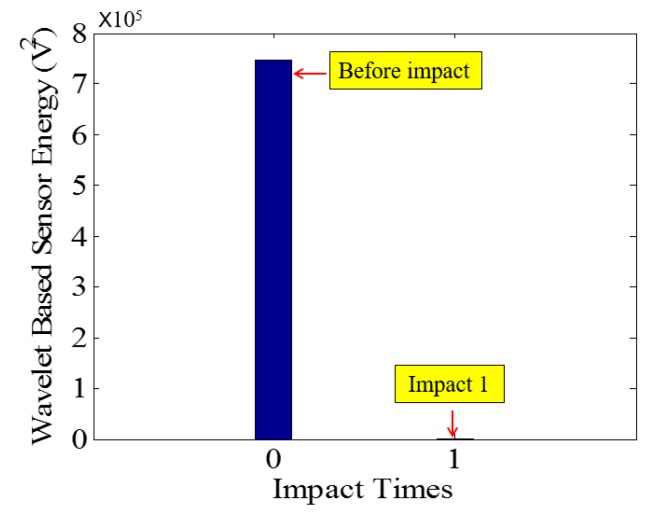
Wavelet packet-based energy plots of the received signal in Mortar-2.

**Figure 25:**
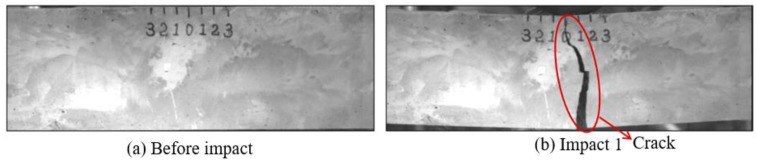
Results of crack detection of Mortar-3 with high-speed video.

**Figure 26:**
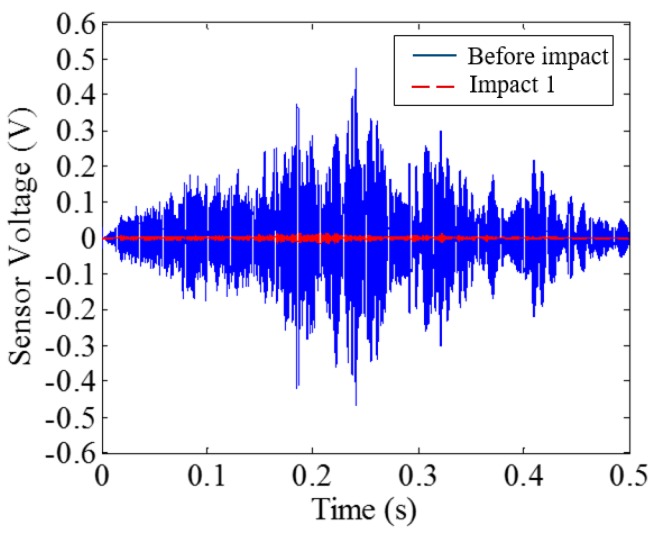
Time-domain signal received by SA sensor in Mortar-2.

**Figure 27:**
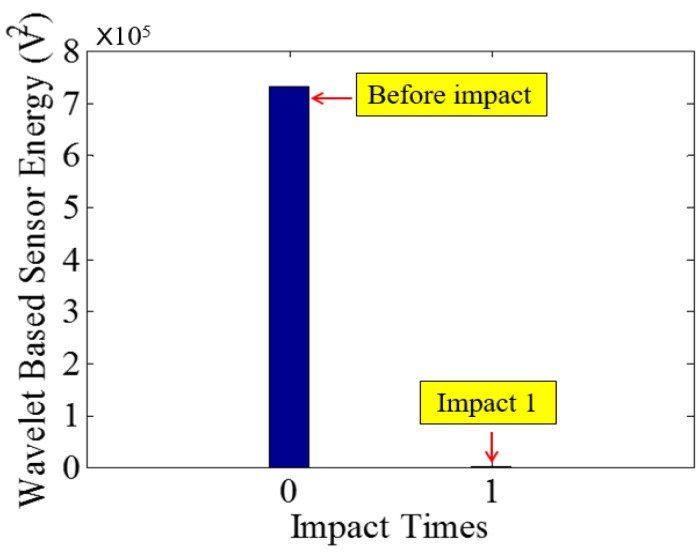
Wavelet packet-based energy plots of the received signal in Mortar-2.

Figure 19, Figure 20, Figure 21, Figure 22, Figure 23, Figure 24, Figure 25, Figure 26 and Figure 27 show the Mortar-1, 2 and 3 beams respectively, after impact tests, the time-domain signal received by the SA sensors, and the signal energy based on wavelet packet-based analysis. Compared to PVA-ECC beams, the drop weight for all mortar beams was 2.724 kg. The only variable was the drop height which decreased from 1.5 to 0.5 m. It was seen that all mortar beams completely failed after the first impact, and no signal response was detected by the SA sensors. Similar results were verified by the wavelet packet-based energy plots. After the first impact test for each beam, the energy of the propagated signal rapidly zeros. 

### 5.3. Discussion

In this research, a combination of PVDF thin-film sensors and smart aggregates were utilized to monitor impact initiation and crack evolution in PVA-ECC and mortar beams. Figure 6, Figure 7, Figure 8 and Figure 9 show that STFT effectively detected impact events and displayed impact information, including impact time, frequency range, and magnitude versus frequency. The bouncing effect was observed and weakened with the increase of impact events. However, since the PVDF thin-film sensors were embedded close to the crack area of the beams, the impact energy detected by the PVDF sensors are influenced by the characteristics of the crack. Therefore, no clear trend of magnitude versus frequency was observed in Figure 6, Figure 7, Figure 8 and Figure 9 from the repeated impact tests. The magnitude vs. frequency information is an important aspect of the results and should be correlated to the structural damage information. For future work, the location of the embedded PVDF thin-film sensors will be optimized to avoid direct effects from the impact-induced crack and the design of the PVDF thin-film sensor will be optimized. Spherical piezoceramic-based transducers might be a solution to eliminate the influence of the crack characteristics to the sensor signal [36,37]. On the other hand, the sampling frequency used in this test was 10 kHz, so the highest computed frequency based on STFT was 5 kHz. The wide frequency response (up to 500 kHz) due to the impact events will be investigated in future work. 

## 6. Conclusions

In this research, the impact damage of PVA-ECC beam under low-velocity impact loading was investigated using PVDF thin-film sensors and smart aggregates. Experimental results show that the PVA-ECC material exhibits improved impact resistance. The impact time, frequency range, and impact energy were obtained by STFT. Experimental results showed that PVA-ECC beams exhibited a bouncing effect under free-fall drop impact tests. In addition, the bouncing effect weakened as the impact-induced crack enlarged. Active sensing using a pair of smart aggregates was successful in monitoring crack evolution in real-time due to multiple repeated impact loadings. As the crack deteriorated, the propagated wave attenuated, and wavelet packet-based energy analysis was able to compute and quantize the sensor energy detected by the smart aggregate sensor. The decreasing trend of the computed sensor energy shows potentials in monitoring the deterioration of cracks in structures under successive impact loadings. For our future work, we will perform more comparative studies of the structural impact behaviors between PVA-ECC and conventional concrete, as well as other commercially available concrete.

## Figures and Tables

**Table 1:** Properties of PVDF and Lead Zirconate Titanate (PZT).

	PVDF	PZT
Density (kg/m^3^)	1800	7750
Compliance (×10^−^^9^ m/N)	0.1	0.02
Relative permittivity	10	1800
*d_33_* (pC/N)	30	410
*d_31_* (pC/N)	‒18	‒175

**Table 2:** Compositions of polyvinyl alcohol fiber reinforced engineering cementitious composite (PVA-ECC) and mortar.

Beam Type	Cement	Sand	Fly Ash	Water	SP (%)	PVA (%)
PVA-ECC	1.16	1.16	2.2	0.66	0.002	2
Mortar	1.16	1.16	2.2	0.66	0.002	0

**Table 3:** Properties of PVA fiber.

Fiber Type	Nominal Strength (MPa)	Apparent Strength (MPa)	Diameter (mm)	Length (mm)	Young’s Modulus (GPa)	Elongation (%)
PVA	1620	1092	39	12	42.8	6.0

**Table 4:** Parameters of the excited swept sine wave signal.

Amplitude (V)	Start Frequency (Hz)	Stop Frequency (kHz)	Period (s)
10	100	250	0.5

**Table 5:** Detailed impact information for each beam specimen.

Specimen	PVA-ECC-1	PVA-ECC-2	PVA ECC-3	Mortar-1	Mortar-2	Mortar-3
Drop weight(kg)	2.724	2.724	8.172	2.724	2.724	2.724
Drop height (m)	1.5	1.0	1.5	1.5	1.0	0.5

**Table 6:** Total number of the impact tests for each beam specimen.

Specimen	PVA-ECC-1	PVA-ECC-2	PVA ECC-3	Mortar-1	Mortar-2	Mortar-3
Number of impact tests	12	15	3	1	1	1
